# Blood Pressure and Angina-Related Health Status During Benidipine Therapy: A Multicenter Real-World Cohort Study

**DOI:** 10.3390/jcm15156047

**Published:** 2026-08-04

**Authors:** Ayşegül Ülgen Kunak, Tolga Kunak, Ertan Aydın, Ümit Yaşar Sinan, Ali Nizami Elmas, Umut Uyan, Berkay Ekici, Onur Aslan, Baran Arık, Kübra Korkmaz, Mustafa Doğduş, Ahmet Ferhat Kaya, Nedret Ülvan, Serhat Çalışkan, Berkant Öztürk, Zafer Kök, Murat Kerkütlüoğlu, Mehmet Kış, Sinan İnci, Mustafa Ahmet Huyut, Vedat Hekimsoy, Emrah Yeşil, Özkan Bekler, Sebahat Tekeli Şengül, Emrah Kaya, Aykut Demirkıran, Sefa Erdi Ömür, Halil İbrahim Durmuş, Şahbender Koç, Saadet Aydın, Fahrettin Katkat, Ömer Kümet, Murat Küçükukur, Aysu Oktay, Mehmet Ertürk, Taner Şen, Nurcemal Şentürk, Hatice Solmaz, Lütfü Bekar, Özkan Kayhan, Müge Ildızlı Demirbaş, Kenan Toprak, Sedat Kanat, Cenk Conkbayır, Alkame Akgümüş, Fahri Er, Ömer Bedir, Ramazan Duz, Harun Recep Narçiçeği, Yasin Deniz, Nezihe İlknur Bostanoğlu, Mehdi Zoghi, Asım Oktay Ergene

**Affiliations:** 1 Department of Cardiology, Antalya Training and Research Hospital, University of Health Sciences, Antalya 07100, Türkiye; 2 Department of Cardiology, Faculty of Medicine, Akdeniz University, Antalya 07100, Türkiye; 3 Department of Cardiology, Giresun Training and Research Hospital, Giresun 28100, Türkiye; 4 Department of Preventive and Epidemiologic Cardiology, Cerrahpasa Cardiology Instutite, Istanbul University, İstanbul 34452, Türkiye; 5 Department of Cardiology, Menderes State Hospital, İzmir 35471, Türkiye; 6 Department of Cardiology, Ödemiş State Hospital, İzmir 35750, Türkiye; 7 Department of Cardiology, Faculty of Medicine, Dr. Rıdvan Ege Hospital, Ufuk University, Ankara 06836, Türkiye; 8 Department of Cardiology, Tarsus State Hospital, Mersin 33440, Türkiye; 9 Department of Cardiology, Torbalı State Hospital, İzmir 35860, Türkiye; 10 Department of Cardiology, Eskişehir City Hospital, Eskişehir 26080, Türkiye; 11 Department of Cardiology, Faculty of Medicine, Medical Point Hospital, İzmir University of Economics, İzmir 35330, Türkiye; 12 Department of Cardiology, Van Training and Research Hospital, University of Health Sciences, Van 65100, Türkiye; 13 Department of Cardiology, Ankara Bilkent City Hospital, Ankara 06800, Türkiye; 14 Department of Cardiology, İstanbul Bahçelievler State Hospital, İstanbul 34186, Türkiye; 15 Department of Cardiology, Faculty of Medicine, Samsun University, Samsun 55080, Türkiye; 16 Department of Cardiology, Sakarya Training and Research Hospital, Sakarya 54100, Türkiye; 17 Department of Cardiology, Faculty of Medicine, Kahramanmaraş Sütçü İmam University, Kahramanmaraş 46040, Türkiye; 18 Department of Cardiology, Faculty of Medicine, Dokuz Eylül University, İzmir 35220, Türkiye; 19 Department of Cardiology, Faculty of Medicine, Aksaray University, Aksaray 68100, Türkiye; 20 Department of Cardiology, Prof. Dr. Cemil Taşcıoğlu City Hospital, İstanbul 34384, Türkiye; 21 Department of Cardiology, Ankara Etlik City Hospital, Ankara 06170, Türkiye; 22 Department of Cardiology, Faculty of Medicine, Mersin University, Mersin 33110, Türkiye; 23 Department of Cardiology, İstanbul Medipol University, İstanbul 34810, Türkiye; 24 Department of Cardiology, Faculty of Medicine, Uşak University, Uşak 64000, Türkiye; 25 Department of Cardiology, Faculty of Medicine, Kutahya Health Sciences University, Kütahya 43020, Türkiye; 26 Department of Cardiology, Faculty of Medicine, Namık Kemal University, Tekirdağ 59030, Türkiye; 27 Department of Cardiology, Faculty of Medicine, Tokat Gazi Osman Paşa University, Tokat 60250, Türkiye; 28 Department of Cardiology, Ankara Sanatoryum Training and Research Hospital, Ankara 06290, Türkiye; 29 Department of Cardiology, Faculty of Medicine, İzmir Bakırçay University, İzmir 35665, Türkiye; 30 Department of Cardiology, İstanbul Training and Research Hospital, İstanbul 34098, Türkiye; 31 Department of Cardiology, İzmir City Hospital, İzmir 35540, Türkiye; 32 Department of Cardiology, Mehmet Akif Ersoy Thoracic And Cardiovascular Surgery Training and Research Hospital, University of Health Sciences, İstanbul 34668, Türkiye; 33 Department of Cardiology, Faculty of Medicine, Karadeniz Technical University, Trabzon 61080, Türkiye; 34 Department of Cardiology, Faculty of Medicine, Hitit University, Çorum 19030, Türkiye; 35 Department of Cardiology, Antalya Kepez State Hospital, Antalya 07320, Türkiye; 36 Department of Cardiology, Koşuyolu Training and Research Hospital, İstanbul 34846, Türkiye; 37 Department of Cardiology, Faculty of Medicine, Harran University, Şanlıurfa 63190, Türkiye; 38 Department of Cardiology, Dr. Burhan Nalbantoğlu State Hospital, Yakın Doğu University, Lefkoşa 99010, Cyprus; 39 Department of Cardiology, Faculty of Medicine, Bandırma 17 Eylül University, Balıkesir 10250, Türkiye; 40 Department of Cardiology, Ağrı Training and Research Hospital, Ağrı 04100, Türkiye; 41 Department of Cardiology, Adana City Training and Research Hospital, Adana 01291, Türkiye; 42 Department of Cardiology, Faculty of Medicine, Yüzüncü Yıl University, Van 65080, Türkiye; 43 Department of Cardiology, Faculty of Medicine, Fırat University, Elazığ 23119, Türkiye; 44 Department of Cardiology, İstanbul Sancaktepe İlhan Varank Training and Research Hospital, İstanbul 34785, Türkiye; 45 Department of Cardiology, Faculty of Medicine, Ege University, İzmir 35040, Türkiye

**Keywords:** hypertension, benidipine, angina pectoris, blood pressure, Seattle Angina Questionnaire, observational study

## Abstract

**Background**: Benidipine is a long-acting calcium channel blocker used for the treatment of hypertension and chronic coronary syndrome. Although it effectively lowers blood pressure (BP), the relationship between BP reduction and improvement in angina-related health status during benidipine therapy remains uncertain. This study evaluated changes in BP and patient-reported angina-related health status in a large real-world cohort. **Methods**: This prospective, multicenter, observational cohort study included 1659 patients with hypertension and/or chronic coronary syndrome who underwent baseline clinical assessment before initiation of benidipine therapy and were reassessed after 3 months. Changes in systolic and diastolic BP and Seattle Angina Questionnaire-7 (SAQ-7) scores were evaluated. Multivariable linear regression analyses were performed to identify predictors of BP reduction and symptomatic improvement. A propensity score-matched analysis according to coronary artery disease (CAD) status was conducted to further explore differences in treatment response. **Results**: The mean age was 62.2 ± 12.2 years, and 54.0% of participants were women. Systolic BP decreased by 13.8 mmHg and diastolic BP by 6.7 mmHg (both *p* < 0.001). Mean SAQ-7 score increased by 8.1 points (*p* < 0.001), exceeding the established threshold for clinically meaningful improvement. Baseline SAQ-7 score showed the strongest association with change in SAQ-7, whereas baseline systolic BP was the strongest predictor of BP reduction. The correlation between changes in systolic BP and SAQ-7 was weak (*r* = 0.058, *p* = 0.023). In the propensity score-matched cohort (n = 732), BP reduction was similar regardless of CAD status, whereas improvement in SAQ-7 was greater in patients without obstructive CAD. **Conclusions**: In this multicenter real-world cohort, benidipine therapy was associated with significant BP reduction and clinically meaningful improvement in angina-related health status over 3 months. Symptomatic improvement showed only a weak association with BP reduction. These findings should be interpreted within the limitations of an observational study and warrant confirmation in controlled comparative studies.

## 1. Introduction

Hypertension remains the most prevalent modifiable risk factor for cardiovascular morbidity and mortality worldwide. Despite advances in pharmacological therapy, blood pressure (BP) control rates remain suboptimal, and many patients continue to experience residual cardiovascular risk even when treated according to current guidelines [1]. These observations highlight the need for comprehensive and individualized treatment strategies.

Contemporary hypertension management extends beyond BP reduction alone and increasingly incorporates symptom burden and patient-reported outcomes. This is particularly relevant in patients with concomitant angina, where symptoms are influenced not only by systemic hemodynamics but also by coronary vasomotor tone, endothelial function, and microvascular circulation.

The Seattle Angina Questionnaire (SAQ) is a validated, disease-specific tool for assessing angina-related health status, including physical limitation, angina frequency, and quality of life [2]. Its shorter version, the Seattle Angina Questionnaire-7 (SAQ-7), maintains strong validity and sensitivity to clinical change, making it suitable for both clinical trials and real-world studies [3]. The Turkish version has also been validated and shown to be reliable in clinical practice [4].

Calcium channel blockers are widely used in patients with hypertension and angina. Benidipine is a long-acting dihydropyridine with unique pharmacological properties, including inhibition of L-, T-, and N-type calcium channels. This profile may result in sustained vasodilation and potential benefits on coronary microvascular function [5]. Beyond its antihypertensive action, benidipine has been associated with improved coronary blood flow, reduced vascular resistance, and anti-inflammatory and antioxidative effects, which may contribute to symptomatic improvement [6,7,8].

Previous studies have demonstrated the antihypertensive and antianginal efficacy of benidipine, particularly in vasospastic angina [9,10,11,12,13]. Whether symptomatic improvement with antihypertensive therapy is primarily driven by blood pressure reduction or by additional vascular effects remains insufficiently understood. Moreover, real-world data integrating hemodynamic outcomes with patient-reported measures are limited.

Therefore, this multicenter, real-world study aimed to evaluate the association of benidipine therapy with changes in BP and angina-related health status assessed by the SAQ-7 in a large, unselected population. In addition, factors associated with symptomatic improvement and BP reduction were explored.

## 2. Materials and Methods

This was a prospective, multicenter, observational, real-world cohort study conducted across 45 cardiology outpatient clinics, including secondary and tertiary care centers such as university hospitals, training and research hospitals, and state hospitals. The study was designed to reflect routine clinical practice.

The primary objective was to evaluate changes in blood pressure and angina-related health status during benidipine therapy over a 3-month follow-up period in patients with hypertension and/or chronic coronary syndrome. Specifically, the study assessed changes in hemodynamic parameters, angina symptom burden, health-related quality of life measured by the SAQ-7, laboratory parameters, and safety outcomes.

Patients were eligible if they were aged between 18 and 80 years, had hypertension and/or angina pectoris, had baseline clinical, laboratory, blood pressure, and SAQ-7 assessments performed before initiation of benidipine therapy, subsequently started benidipine according to the treating physician’s decision in routine clinical practice, and completed the scheduled 3-month follow-up evaluation. Benidipine dosing (4 mg vs. 8 mg) was determined by the treating physician based on clinical judgment, symptom burden, blood pressure control, and routine practice patterns. Continued benidipine therapy during follow-up was planned in all included patients.

Patients were excluded if they had secondary hypertension, hypertensive emergency, or required initiation of additional therapies during follow-up that could significantly affect blood pressure. Other exclusion criteria included known hypersensitivity to dihydropyridine calcium channel blockers, decompensated heart failure, unstable angina, clinically significant arrhythmias, severe renal or hepatic dysfunction, pregnancy or breastfeeding, participation in another clinical trial within the previous three months, or failure to provide informed consent.

A total of 1659 patients were enrolled in the study. All patients completed the 3-month follow-up. Analyses were performed using available-case data. Missing data were infrequent: baseline BP (<1%), SAQ-7 (<2%), laboratory variables (0.5–4.2%), and follow-up BP and SAQ-7 (<2%). Because the proportion of missing data for all variables was below 5%, no imputation was performed.

Baseline data included demographic characteristics, cardiovascular risk factors, comorbidities, medication use, laboratory findings, and hemodynamic measurements. Hypertension was defined as office systolic BP ≥140 mmHg and/or diastolic BP ≥90 mmHg, or current use of antihypertensive medication, in accordance with contemporary guideline recommendations. Chronic kidney disease was defined as an estimated glomerular filtration rate <60 mL/min/1.73 m^2^. Coronary artery disease (CAD) was defined as ≥50% stenosis in at least one major epicardial coronary artery. Angina severity was classified using the Canadian Cardiovascular Society (CCS) grading system.

Blood pressure was measured at each participating outpatient clinic according to routine clinical practice. Measurements were obtained in the seated position after at least 5 min of rest using validated automated or calibrated sphygmomanometers available at each center. When more than one measurement was obtained at a study visit, the average value was used for analysis.

Angina-related health status was assessed using the SAQ-7. Scores were transformed to a standardized 0–100 scale, with higher scores indicating better health status. Change in angina-related health status (ΔSAQ-7) was defined as the difference between baseline and 3-month values.

Patients were evaluated at baseline and at 3 months. The primary outcomes were the change in systolic blood pressure (ΔSBP) and the change in SAQ-7. Secondary outcomes included changes in diastolic blood pressure, laboratory parameters, CCS angina class, nitroglycerin use, and safety outcomes, including adverse events and treatment discontinuation. Treatment continuation at follow-up was assessed based on patient self-report and outpatient medication records.

### Statistical Analysis

Continuous variables are presented as mean ± standard deviation or median (interquartile range), as appropriate, while categorical variables are expressed as number and percentage. Comparisons between baseline and follow-up were performed using paired *t*-tests or Wilcoxon signed-rank tests for continuous variables and χ^2^ tests for categorical variables, as appropriate.

Multivariable linear regression analyses were conducted to identify independent predictors of ΔSAQ-7 and ΔSBP. Clinically relevant variables were included regardless of univariable significance. Multicollinearity was assessed using variance inflation factors. Results are presented as regression coefficients (B), standardized coefficients (β), and 95% confidence intervals (CI). A two-sided *p*-value <0.05 was considered statistically significant.

To further explore the relationship between hemodynamic improvement and symptomatic benefit, the correlation between ΔSBP and ΔSAQ-7 was assessed using Pearson correlation analysis.

To minimize the impact of potential selection bias and confounding inherent to the observational design, a propensity score-matched (PSM) analysis was performed. Propensity score matching was conducted according to CAD status to explore whether changes in angina-related health status differed between patients with and without obstructive CAD. Because all patients received benidipine, the propensity score analysis was intended to balance baseline differences between the CAD groups rather than estimate the treatment effect of benidipine itself. The following baseline covariates were selected based on their clinical relevance: age, sex, baseline systolic blood pressure, baseline SAQ-7 score, heart rate, smoking status, diabetes mellitus, beta-blocker use, and benidipine dose.

Patients with and without obstructive CAD were matched in a 1:1 ratio using nearest-neighbor matching without replacement and a predefined caliper width of 0.02 of the standard deviation of the logit of the propensity score. Covariate balance after matching was assessed using standardized mean differences (SMD), with a value <0.1 considered indicative of adequate balance. In the matched cohort, continuous variables were compared using paired or independent t-tests as appropriate, and categorical variables using χ^2^ tests. Changes in outcomes between groups were compared to evaluate differences in treatment response according to CAD status.

No formal adjustment for multiple comparisons was applied, as the analyses were planned a priori and interpreted in the context of their clinical relevance.

**Figure 1:**
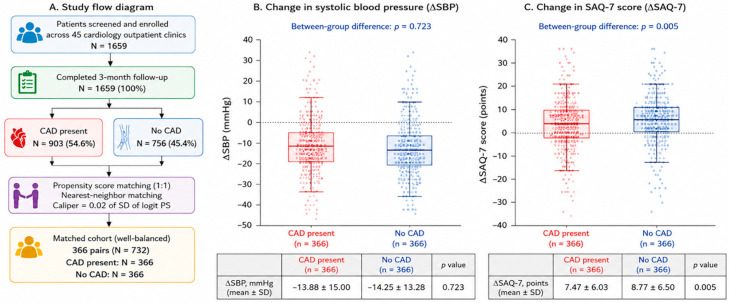
Study flow and changes in systolic blood pressure and angina-related health status in the propensity score–matched cohort. (**A**) Flow diagram illustrating patient enrollment and 1:1 propensity score matching according to coronary artery disease (CAD) status. (**B**) Distribution of the change in systolic blood pressure (ΔSBP) in the matched CAD and non-CAD groups. (**C**) Distribution of the change in Seattle Angina Questionnaire-7 (ΔSAQ-7) score in the matched CAD and non-CAD groups. Between-group comparisons in panels (**B**,**C**) were performed using the independent-samples *t* test. Boxes represent the interquartile range (IQR), the horizontal line indicates the median, whiskers represent 1.5 × IQR, and dots represent individual observations.

All analyses were performed using IBM SPSS Statistics version 27.0 (IBM Corp., Armonk, NY, USA). The patient selection process and the details of the propensity score-matching procedure, which resulted in 366 well-balanced pairs, are summarized in Figure 1A.

## 3. Results

### 3.1. Baseline Characteristics

A total of 1659 patients were included in the study. The mean age was 62.2 ± 12.2 years, and 54.0% were female. Hypertension was present in 93.8% of patients, including 37.6% newly diagnosed cases. CAD was documented in 54.6% of the cohort. Baseline systolic and diastolic blood pressures were 142.4 ± 14.9 mmHg and 84.4 ± 9.6 mmHg, respectively. Mean left ventricular ejection fraction was 57.5 ± 6.9%. Most patients had mild-to-moderate angina, with 67.6% in CCS class I–II, and 73.9% did not require nitroglycerin at baseline. Baseline characteristics of the study population are summarized in Table 1.

At baseline, 42.6% of patients were receiving benidipine 4 mg/day and 57.1% were receiving 8 mg/day. At 3 months, the proportion receiving 8 mg/day increased to 61.7%, while 38.1% remained on 4 mg/day. Concomitant therapies included renin–angiotensin system blockers, beta-blockers, statins, and antianginal agents such as ranolazine (15.8%), ivabradine (4.7%), and trimetazidine (10.7%). Concomitant antihypertensive and antianginal medications remained largely unchanged during follow-up, except for benidipine dose adjustment according to routine clinical practice (Supplementary Table S1).

### 3.2. Changes in Blood Pressure and Laboratory Findings

Systolic blood pressure significantly decreased from 142.4 ± 14.9 mmHg at baseline to 128.6 ± 11.6 mmHg at 3 months (Δ −13.8 mmHg, *p* < 0.001). Diastolic blood pressure decreased from 84.4 ± 9.6 mmHg to 77.6 ± 7.9 mmHg (Δ −6.7 mmHg, *p* < 0.001). The reduction in systolic blood pressure was consistent across dose groups (−13.3 mmHg with 4 mg and −14.5 mmHg with 8 mg), with no significant between-group difference. Changes in hemodynamic and laboratory parameters are presented in Table 2.

Heart rate remained stable during follow-up (75.7 ± 10.1 vs. 75.6 ± 10.6 bpm, *p* = 0.06). Renal function was unchanged (median eGFR 73.7 vs. 73.0 mL/min/1.73 m^2^, *p* = 0.45). Small reductions were observed in fasting glucose and LDL cholesterol during follow-up, whereas serum creatinine, hemoglobin, and hs-CRP remained unchanged.

### 3.3. Changes in Angina-Related Health Status

Angina severity improved over 3 months. The proportion of patients in CCS class I increased from 39.2% to 51.0%, whereas CCS class III decreased from 11.1% to 3.6%. The proportion of patients not requiring nitroglycerin increased from 73.9% to 79.9% (*p* < 0.001). Changes in angina-related outcomes are summarized in Table 3. Over the 3-month follow-up, SAQ-7 scores increased by a mean of 8.08 ± 6.27 points (*p* < 0.001), exceeding the established minimal clinically important difference threshold of 5 points for the SAQ-7.

### 3.4. Predictors of Treatment Response

Baseline SAQ-7 score was the strongest independent predictor of improvement in SAQ-7 (β = −0.685, *p* < 0.001), followed by CCS class (β = −0.164, *p* < 0.001). In the multivariable model, higher benidipine dose showed a small statistical association with greater improvement in SAQ-7 (β = 0.045, *p* = 0.033), whereas beta-blocker use was associated with less improvement (β = −0.047, *p* = 0.037).

For systolic blood pressure reduction, baseline SBP was the strongest predictor (β = 0.707, *p* < 0.001), followed by diabetes, CCS class, and age. Benidipine dose was not independently associated with ΔSBP. Multivariable regression analyses are presented in Table 4a,b.

The correlation between systolic blood pressure reduction and improvement in angina-related health status was very weak (r = 0.058, *p* = 0.023).

### 3.5. Propensity Score-Matched Analysis

To further explore the impact of CAD status on treatment response, a propensity score-matched analysis was performed. A total of 366 matched pairs (N = 732) were identified, with well-balanced baseline characteristics. Standardized mean differences after matching ranged from 0.000 to 0.073, indicating good covariate balance (Supplementary Table S2). In the matched cohort, the reduction in systolic blood pressure was comparable between patients with and without CAD (−13.88 ± 15.00 vs. −14.25 ± 13.28 mmHg, *p* = 0.723) (Figure 1B). However, the improvement in angina-related health status was significantly greater in patients without CAD compared to those with CAD (ΔSAQ-7: 8.77 ± 6.50 vs. 7.47 ± 6.03, *p* = 0.005) (Figure 1C). Results of the propensity score-matched analysis are shown in Table 5.

### 3.6. Safety and Clinical Outcomes

During the 3-month follow-up, peripheral edema occurred in 4.3% of patients, headache in 6.0%, dizziness in 4.8%, and hypotension in 3.6%. Overall, 92.3% of patients remained on benidipine therapy at the 3-month follow-up. Detailed adverse events are provided in Supplementary Table S3.

Cardiovascular hospitalization occurred in 5.5% of patients. Acute coronary syndrome was observed in 1.3%, and stroke in 0.5%. Additional clinical events are summarized in Supplementary Table S4.

## 4. Discussion

This large, multicenter, real-world cohort study showed that benidipine therapy was associated with significant reductions in both systolic and diastolic blood pressure, along with clinically meaningful improvements in angina-related health status over a 3-month period. The magnitude of blood pressure reduction observed in this study (−13.8/−6.7 mmHg) was clinically relevant and consistent with contemporary evidence indicating that even modest reductions in blood pressure are associated with substantial cardiovascular risk reduction [1,14,15].

In addition to blood pressure reduction, one of the most notable findings is the improvement in angina-related health status, with an approximately 8-point increase in SAQ-7 score. The approximately 8-point increase observed in SAQ-7 score exceeded the established minimal clinically important difference threshold of 5 points, indicating that the observed benefit was not only statistically significant but also clinically perceptible from a patient-centered perspective because patients would be expected to experience a meaningful improvement in their symptom burden and daily functioning [2,3,16].

A key observation is that baseline symptom burden appeared to be more strongly associated with improvement in angina-related health status than blood pressure reduction. Baseline SAQ-7 score showed the strongest statistical association with change in SAQ-7. This finding should be interpreted cautiously because analyses based on change scores may partly reflect regression to the mean. This finding is consistent with prior studies evaluating patient-reported outcomes, where baseline disease severity is a major determinant of the magnitude of improvement [3,16]. Supporting this observation, the correlation between ΔSBP and ΔSAQ-7, although statistically significant, was negligible in magnitude (r = 0.058). Given the large sample size, this statistical significance should not be interpreted as evidence of a clinically meaningful relationship, suggesting that reductions in systemic blood pressure alone are unlikely to fully explain the observed symptomatic benefit.

Interestingly, benidipine dose was independently associated with symptomatic improvement but not with systolic blood pressure reduction. Although benidipine dose was associated with greater improvement in SAQ-7, this finding should be interpreted cautiously because dose selection was not randomized and may reflect confounding by indication. Benidipine inhibits L-, T-, and N-type calcium channels, and previous experimental and clinical studies have suggested favorable effects on coronary vasomotor function, endothelial function, inflammation, and oxidative stress [5,17,18,19]. Experimental studies have shown that T-type calcium channel inhibition has been associated with suppression of sympathetic activation and improvement in coronary microcirculatory resistance, whereas N-type blockade may reduce neurohumoral activation and vasoconstrictive responses. These experimentally supported pharmacological properties provide a biologically plausible explanation for our findings; however, the present study did not evaluate coronary vasomotor function, endothelial function, or microvascular physiology. Therefore, the present study cannot determine whether these mechanisms contributed to the observed symptomatic improvement cannot be determined from the current observational data.

These mechanisms may be particularly relevant in patients with microvascular dysfunction or vasospastic angina, where symptoms are not directly proportional to epicardial coronary stenosis [20,21,22]. Because detailed coronary functional testing was not available, the potential contribution of microvascular dysfunction or vasospastic mechanisms remains speculative.

In the propensity score-matched cohort, improvement in SAQ-7 was modestly greater in patients without obstructive CAD despite similar BP reduction. Although the absolute between-group difference was small, this observation raises the possibility that treatment response may differ according to underlying coronary phenotype. However, because propensity score matching balanced baseline characteristics only between CAD phenotypes and all patients received benidipine, this analysis cannot eliminate treatment-selection bias or establish differential treatment effects.

Our findings are consistent with previous clinical and real-world studies evaluating benidipine. However, in the absence of a control group, the present study cannot distinguish drug-specific effects from class effects of calcium channel blockers. Prior investigations have demonstrated that benidipine reduces angina frequency and improves clinical outcomes in both stable and vasospastic angina populations [11,12,23]. Comparative studies suggest that benidipine may offer similar or, in some contexts, favorable efficacy compared with other calcium channel blockers such as amlodipine or diltiazem [10,24]. In current clinical practice, amlodipine is generally the preferred long-acting dihydropyridine calcium channel blocker for hypertension, whereas benidipine is more commonly used in several Asian countries, particularly Japan, where evidence has suggested potential benefits in patients with vasospastic angina and microvascular dysfunction. In addition, long-term studies have suggested potential benefits in cardiovascular and renal outcomes [25,26]. The present study extends this evidence by integrating hemodynamic effects with patient-reported outcomes in a large, unselected population.

Another notable finding is the inverse association between beta-blocker use and improvement in angina-related health status. This observation should be interpreted cautiously and is most likely explained by confounding by indication, as patients receiving beta-blockers may have had more severe or refractory symptoms at baseline. However, it may also reflect differences in therapeutic response across angina phenotypes, as calcium channel blockers are generally more effective in vasospastic and microvascular angina, whereas beta-blockers may be less beneficial in these subgroups [27].

From a hemodynamic perspective, predictors of systolic blood pressure reduction were largely driven by baseline blood pressure levels, with additional contributions from age, diabetes, and angina severity. The lack of an independent association between benidipine dose and ΔSBP suggests that dose escalation within the studied range may have limited incremental impact on blood pressure reduction. At the same time, clinicians may preferentially increase dose in more symptomatic patients, which could partially explain the observed association with symptomatic improvement.

The multivariable model explained a moderate proportion of variability in ΔSAQ-7, indicating that a substantial proportion of variability remains unexplained. Factors such as coronary microvascular dysfunction, endothelial function, and psychosocial determinants may contribute but were not captured in the present analysis.

The incidence of adverse events was low and consistent with the known safety profile of dihydropyridine calcium channel blockers, including headache, dizziness, and peripheral edema [9,10,24]. Importantly, the rate of peripheral edema remained modest and comparable across dose groups. Although causal inferences cannot be drawn from this observational study, no new safety concerns were identified, and the observed tolerability profile was consistent with previous reports of benidipine.

The weak association between BP reduction and symptom improvement suggests that patient-reported outcomes may provide additional clinical information beyond hemodynamic measures alone. The use of SAQ-7 provides a comprehensive assessment of treatment benefit and aligns with the increasing emphasis on patient-centered care in cardiovascular medicine [28,29]. Accordingly, part of the observed improvement in SAQ-7 may reflect non-pharmacological influences inherent to longitudinal observational studies, including regression to the mean and increased clinical attention during follow-up, rather than the pharmacological effects of benidipine alone.

Several limitations should be acknowledged. First, the observational design precludes causal inference. Second, residual confounding cannot be excluded despite multivariable adjustment and propensity score matching. Third, the relatively short follow-up limits assessment of long-term outcomes. Fourth, because benidipine dose selection was not randomized and was determined by physician preference, confounding by indication cannot be excluded. Fifth, treatment adherence was not objectively verified and partly relied on self-reported medication use. Sixth, the lack of detailed phenotyping of angina (e.g., vasospastic versus microvascular) limits mechanistic interpretation. Finally, because all patients received benidipine, regression to the mean, placebo effects, and increased clinical attention during follow-up may also have contributed to the observed improvements.

## 5. Conclusions

In this prospective multicenter real-world cohort, benidipine therapy was associated with clinically meaningful improvement in angina-related health status together with significant blood pressure reduction. The weak correlation between BP reduction and improvement in SAQ-7 suggests that symptomatic and hemodynamic changes were not closely related in this cohort. Benidipine was also well tolerated during the 3-month follow-up. These findings should be interpreted within the limitations of the observational study design. Because this study lacked a comparator group, no conclusions can be drawn regarding the superiority or specific effects of benidipine compared with other calcium channel blockers. Further controlled studies are needed to determine whether these observations are attributable to benidipine and whether treatment response differs according to coronary disease phenotype.

## Figures and Tables

**Table 1:** Baseline clinical and demographic characteristics of the study population.

Variable	Total (N = 1659)
Age, years	62.23 ± 12.19
BMI, kg/m^2^	27.83 ± 3.68
Systolic BP, mmHg	142.40 ± 14.92
Diastolic BP, mmHg	84.38 ± 9.60
LVEF, %	57.53 ± 6.89
Serum creatinine, mg/dL	0.96 ± 0.33
Hemoglobin, g/dL	13.18 ± 1.68
Glucose, mg/dL	111.06 ± 36.94
LDL cholesterol, mg/dL	111.07 ± 34.25
hs-CRP, mg/L	3 (1.9–5.7)
HbA1c, %	6.51 ± 1.32
Female sex	895 (54.0)
Hypertension	1556 (93.8)
Newly diagnosed HT	623 (37.6)
Coronary artery disease	903 (54.6)
Diabetes mellitus	508 (30.7)
Smoking (current)	556 (33.6)
Hyperlipidemia	855 (51.6)
History of stroke	138 (8.3)
Chronic kidney disease	221 (13.3)

Data are presented as mean ± standard deviation, median (interquartile range), or number (percentage), as appropriate. BMI: body mass index; BP: blood pressure; LVEF: left ventricular ejection fraction; hs-CRP: high-sensitivity C-reactive protein.

**Table 2:** Changes in hemodynamic and laboratory parameters from baseline to 3 months.

Variable	Baseline	3 Months	*p*-Value
SBP (mmHg)	142.40 ± 14.92	128.57 ± 11.63	<0.001
DBP (mmHg)	84.38 ± 9.60	77.64 ± 7.93	<0.001
Heart rate (beats/min)	75.73 ± 10.13	75.65 ± 10.55	0.06
Fasting Glucose (mg/dL)	111.06 ± 36.94	108.22 ± 34.01	<0.001
LDL cholesterol (mg/dL)	111.07 ± 34.25	104.14 ± 31.77	<0.001
Creatinine (mg/dL)	0.96 ± 0.33	0.96 ± 0.29	0.45
Hemoglobin, g/dL	13.18 ± 1.68	13.08 ± 1.54	0.73
hs-CRP, mg/L	3 (1.9–5.7)	3 (1.6–6.2)	0.35

Data are presented as mean ± standard deviation or median (interquartile range). BP: blood pressure; SBP: systolic blood pressure; DBP: diastolic blood pressure.

**Table 3:** Changes in angina severity, symptom burden, and health status.

Variable	Baseline	3 Months	*p*-Value
SAQ-7 score (points)	86.8 ± 10.6	94.9 ± 8.9	<0.001
CCS class I (%)	39.2	51.0	<0.001
CCS class II (%)	28.4	16.9	<0.001
CCS class III (%)	11.1	3.6	<0.001
CCS class IV (%)	2.1	1.6	0.08
No angina (%)	19.3	21.3	0.06
Nitroglycerin-free (%)	73.9	79.9	<0.001

Data are presented as mean ± standard deviation or percentage. SAQ-7: Seattle Angina Questionnaire-7; CCS: Canadian Cardiovascular Society.

**Table 4:** (**a**) Multivariable linear regression analysis for change in systolic blood pressure (ΔSBP). (**b**) Multivariable linear regression analysis for change in angina-related health status (ΔSAQ-7).

**(a)**			
**Variable**	**B (95% CI)**	**β**	***p*-Value**
Baseline SBP (mmHg)	0.676 (0.640 to 0.711)	0.707	<0.001
Age (years)	0.044 (0.002 to 0.086)	0.038	0.041
Sex (female)	0.296 (−0.711 to 1.304)	0.011	0.564
Diabetes mellitus	−2.393 (−3.526 to −1.261)	−0.079	<0.001
Baseline CCS class	−2.093 (−2.633 to −1.553)	−0.143	<0.001
Benidipine dose, mg	−0.181 (−0.437 to 0.075)	−0.026	0.165
ACE inhibitor/ARB use	0.156 (−0.910 to 1.222)	0.005	0.775
**(b)**			
**Variable**	**B (95% CI)**	**β**	***p*** **-Value**
Baseline SAQ-7 score (points)	−0.350 (−0.374 to −0.325)	−0.685	<0.001
Age (years)	−0.006 (−0.029 to 0.016)	−0.012	0.580
Sex (female)	0.036 (−0.490 to 0.562)	0.003	0.893
Benidipine dose (mg)	0.144 (0.012 to 0.276)	0.045	0.033
Baseline CCS class	−1.121 (−1.447 to −0.796)	−0.164	<0.001
Ranolazine use	0.541 (−0.227 to 1.309)	0.032	0.167
Ivabradine use	−0.321 (−1.523 to 0.882)	−0.011	0.601
Beta-blocker use	−0.642 (−1.245 to −0.039)	−0.047	0.037
Diabetes mellitus	−0.108 (−0.703 to 0.486)	−0.008	0.721
Baseline Systolic BP (mmHg)	−0.009 (−0.027 to 0.009)	−0.021	0.321
Trimetazidine use	−0.335 (−1.205 to 0.535)	−0.016	0.450

ACE, angiotensin-converting enzyme; ARB, angiotensin receptor blocker; B, unstandardized regression coefficient; β, standardized regression coefficient; BP, blood pressure; CCS, Canadian Cardiovascular Society; CI, confidence interval; SAQ-7, Seattle Angina Questionnaire-7; SBP, systolic blood pressure.

**Table 5:** Changes in outcomes according to coronary artery disease status after propensity score matching.

Outcome	CAD− (n = 366)	CAD+ (n = 366)	*p*-Value
ΔSAQ-7 score, points	8.77 ± 6.50	7.47 ± 6.03	0.005
ΔSBP, mmHg	−14.25 ± 13.28	−13.88 ± 15.00	0.723

Values are presented as mean ± standard deviation. Δ indicates change from baseline to 3 months. CAD: coronary artery disease; SBP: systolic blood pressure.

## Data Availability

The datasets used and analyzed during the current study are available from the corresponding author on reasonable request.

## Supplementary Materials

The following supporting information can be downloaded at https://www.mdpi.com/article/10.3390/jcm15156047/s1, Table S1: Concomitant cardiovascular medications at baseline; Table S2: Baseline characteristics after propensity score matching; Table S3: Adverse events during the 3-month follow-up; Table S4: Clinical Events During Follow-up.

## Abbreviations

The following abbreviations are used in this manuscript:

ACEI	Angiotensin-Converting Enzyme Inhibitor
ARB	Angiotensin Receptor Blocker
BP	Blood Pressure
CAD	Coronary Artery Disease
CCS	Canadian Cardiovascular Society
CI	Confidence Interval
DBP	Diastolic Blood Pressure
eGFR	Estimated Glomerular Filtration Rate
hs-CRP	High-Sensitivity C-Reactive Protein
LDL	Low-Density Lipoprotein Cholesterol
LVEF	Left Ventricular Ejection Fraction
PSM	Propensity Score Matching
SAQ-7	Seattle Angina Questionnaire-7
SBP	Systolic Blood Pressure
SMD	Standardized Mean Difference

## References

[1] McEvoy J.W., McCarthy C.P., Bruno R.M., Brouwers S., Canavan M.D., Ceconi C., Christodorescu R.M., Daskalopoulou S.S., Ferro C.J., Gerdts E. (2024). 2024 ESC Guidelines for the management of elevated blood pressure and hypertension. Eur. Heart J..

[2] Spertus J.A., Winder J.A., Dewhurst T.A., Deyo R.A., Prodzinski J., McDonell M. (1995). Development and evaluation of the Seattle Angina Questionnaire: A new functional status measure for coronary artery disease. J. Am. Coll. Cardiol..

[3] Chan P.S., Jones P.G., Arnold S.A., Spertus J.A. (2014). Development and validation of a short version of the Seattle Angina Questionnaire. Circ. Cardiovasc. Qual. Outcomes.

[4] Duruöz M.T., Şanal Toprak C., Ulutatar F., Suhaimi A., Agirbasli M. (2020). The validity and reliability of the Turkish version of the Seattle Angina Questionnaire. Turk. Kardiyol. Dern. Ars..

[5] Yao K., Nagashima K., Miki H. (2006). Pharmacological, pharmacokinetic, and clinical properties of benidipine hydrochloride, a novel, long-acting calcium channel blocker. J. Pharmacol. Sci..

[6] Liao P., Yong T.F., Liang M.C., Yue D.T., Soong T.W. (2005). Splicing for alternative structures of Cav1.2 Ca^2+^ channels in cardiac and smooth muscles. Cardiovasc. Res..

[7] Servi H., Korkmaz T.B., Ayaz F. (2024). Anti-inflammatory activity of benidipine hydrochloride in LPS-activated mammalian macrophages. Naunyn Schmiedebergs Arch. Pharmacol..

[8] Suzuki O., Yoshida T., Tani S., Kato K., Yoneyama A., Hibino T., Yokoi K., Matsubara T. (2004). Antioxidative effects of benidipine hydrochloride in patients with hypertension independent of antihypertensive effects. Arzneimittelforschung.

[9] Ohta M., Sugawara S., Sato N., Kuriyama C., Hoshino C., Kikuchi A. (2009). Effects of benidipine, a long-acting T-type calcium channel blocker, on home blood pressure and renal function in patients with essential hypertension: A retrospective, ‘real-world’ comparison with amlodipine. Clin. Drug Investig..

[10] Jadhav U., Mohanan P.P., Almeida A.F., Abraham G., Khan M.Y., Gaurav K., Mane A., Vikas S., Jain M., Meel B. (2021). Effectiveness and effect on renal parameters of amlodipine vs. other dihydropyridine CCBs. Cardiol. Ther..

[11] Suzuki H., Yokoyama K., Akimoto Y., Daida H. (2007). Clinical efficacy of benidipine for vasospastic angina pectoris. Arzneimittelforschung.

[12] Fukumoto Y., Yasuda S., Ito A., Shimokawa H. (2008). Prognostic effects of benidipine in vasospastic angina. J. Cardiovasc. Pharmacol..

[13] Oikawa Y., Matsuno S., Yajima J., Nakamura M., Ono T., Ishiwata S., Fujimoto Y., Aizawa T. (2010). ATTACK CSA trial. J. Cardiol..

[14] Jones D.W., Ferdinand K.C., Taler S.J., Johnson H.M., Shimbo D., Abdalla M., Altieri M.M., Bansal N., Bello N.A., Bress A.P. (2025). 2025 AHA/ACC guideline for hypertension. Hypertension.

[15] Ettehad D., Emdin C.A., Kiran A., Anderson S.G., Callender T., Emberson J., Chalmers J., Rodgers A., Rahimi K. (2016). Blood pressure lowering for prevention of cardiovascular disease. Lancet.

[16] Thomas M., Jones P.G., Arnold S.V., Spertus J.A. (2021). Interpretation of Seattle Angina Questionnaire. JAMA Cardiol..

[17] Nakagomi A., Kodani E., Takano H., Uchida T., Sato N., Ibuki C., Kusama Y., Seino Y., Munakata K., Mizuno K. (2011). Calcium antagonist for ischemic heart attack. Circ. J..

[18] Matsubara M., Yao K., Hasegawa K. (2006). Benidipine inhibits endothelial injury via NO release. Pharmacol. Res..

[19] Mordi I., Mordi N., Delles C., Tzemos N. (2016). Endothelial dysfunction in hypertension. J. Hypertens..

[20] Ford T.J., Berry C. (2019). How to Diagnose and Manage Angina Without Obstructive Coronary Artery Disease: Lessons from the British Heart Foundation CorMicA Trial. Interv. Cardiol..

[21] Kunadian V., Chieffo A., Camici P.G., Berry C., Escaned J., Maas A.H., Prescott E., Karam N., Appelman Y., Fraccaro C. (2021). An EAPCI Expert Consensus Document on Ischaemia with Non-Obstructive Coronary Arteries in Collaboration with European Society of Cardiology Working Group on Coronary Pathophysiology & Microcirculation Endorsed by Coronary Vasomotor Disorders International Study Group. EuroIntervention.

[22] Taqueti V.R., Di Carli M.F. (2018). Coronary microvascular disease. J. Am. Coll. Cardiol..

[23] Kim S.E., Jo S.-H., Han S.H., Lee K.Y., Her S.H., Lee M.-H., Seo W.-W., Cho S.-S., Baek S.H. (2021). Calcium-channel blockers in vasospastic angina. Korean J. Intern. Med..

[24] Sofy A.A., Abdelsattar A.T., Mohammed O.M., Shareef M.A., Alamodi A.A., Nso N., Payton M., Masoud A.T. (2020). Amlodipine vs benidipine meta-analysis. High. Blood Press. Cardiovasc. Prev..

[25] Umemoto S., Ogihara T., Matsuzaki M., Rakugi H., Ohashi Y., Saruta T., Combination Therapy of Hypertension to Prevent Cardiovascular Events (COPE) Trial Group (2017). Effects of calcium channel blocker benidipine-based combination therapy on target blood pressure control and cardiovascular outcome: A sub-analysis of the COPE trial. Hypertens. Res..

[26] Umemoto S., Ogihara T., Matsuzaki M., Rakugi H., Shimada K., Hayashi K., Makino H., Ohashi Y., Saruta T. (2020). Antihypertensive combination and vascular events. Curr. Hypertens. Rev..

[27] Bangalore S., Steg G., Deedwania P., Crowley K., Eagle K.A., Goto S., Ohman E.M., Cannon C.P., Smith S.C., Zeymer U. (2012). β-blocker use outcomes. JAMA.

[28] Biondi-Zoccai G., Frati G., Peruzzi M., Bernardi M., Spadafora L., Tremoli E. (2025). PRO(M)s in cardiovascular research. J. Cardiovasc. Pharmacol..

[29] Spertus J.A., Singh A.T., Arnold S.V. (2024). Future of patient-reported outcomes. Circ. Cardiovasc. Qual. Outcomes.

