# Supplementary material for: Blood Pressure and Angina-Related Health Status During Benidipine Therapy: A Multicenter Real-World Cohort Study
Source: J Clin Med. 2026 Aug 4;15(15):6047. doi: 10.3390/jcm15156047 (PMC13466924; doi:10.3390/jcm15156047)
Supplement: Supplementary file 1 [file jcm-15-06047-s001.zip › jcm-4445193-supplementary.pdf]

SUPPLEMENTARY TABLES

Supplementary Table S1. Concomitant cardiovascular medications at baseline

**Medication**	**n (%)**
**ACE inhibitor**	**323 (19.5)**
**ACE inhibitor + diuretic**	**196 (11.8)**
**ARB**	**234 (14.1)**
**ARB + diuretic**	**330 (19.9)**
**Beta Blocker**	**505 (30.4)**
**Ranolazine**	**261 (15.8)**
**Ivabradine**	**78 (4.7)**
**Mineralocorticoid receptor antagonist**	**155 (9.4)**
**Diuretic (monotherapy)**	**208 (12.6)**
**Alpha-blocker**	**137 (8.3)**
**Aspirin**	**793 (47.9)**
**Clopidogrel**	**272 (16.4)**
**Ticagrelor**	**81 (4.9)**
**Prasugrel**	**16 (1.0)**
**Warfarin**	**83 (5.0)**
**NOAC (any)**	**178 (10.7)**
**Statin**	**831 (50.2)**
**Statin + ezetimibe**	**72 (4.3)**
**Oral antidiabetic drugs**	**447 (27.0)**

Medication	n (%)
**Colchicine**	**177 (10.7)**
**Trimetazidine**	**177 (10.7)**

Data are presented as number (percentage). Percentages are calculated based on available data.

Supplementary Table S2. Baseline characteristics after propensity score matching

Variable	CAD− (n=366)	CAD+ (n=366)	SMD
**Age, years**	61.86 ± 12.45	62.29 ± 12.20	0.035
**Systolic BP, mmHg**	142.68 ± 13.90	142.68 ± 13.58	0.000
**Baseline SAQ-7 score**	86.61 ± 10.69	87.12 ± 10.44	0.048
**Heart rate, bpm**	76.17 ± 10.50	76.34 ± 9.55	0.017
**Male sex, %**	44.5	43.4	0.022
**Smoking, %**	51.6	51.1	0.010
**Diabetes mellitus, %**	29.2	28.4	0.018
**Beta-blocker use, %**	24.3	27.6	0.073
**High-dose benidipine, %**	60.9	60.1	0.017

Values are presented as mean ± standard deviation or percentage. SMD: standardized mean difference. A value <0.1 indicates adequate balance between groups.

Supplementary Table S3. Adverse events during the 3-month follow-up

Adverse event	n (%)
Hypotension	59 (3.6)
Peripheral (ankle) edema	71 (4.3)
Headache	100 (6.0)
Dizziness	80 (4.8)
Flushing	43 (2.6)
Constipation	22 (1.3)
Pruritus	80 (4.8)
Skin rash	15 (0.9)
Photosensitivity	10 (0.6)
Dyspnea	16 (1.0)
Other adverse events	8 (0.5)

Data are presented as number (percentage). Percentages are calculated based on available data.

Supplementary Table S4. Clinical Events During Follow-up

**Clinical event**	**n (%)**
Hospitalization due to cardiovascular causes	92 (5.5)
New acute coronary syndrome	22 (1.3)
Hypertension-related events	28 (1.7)
New stroke	8 (0.5)

Data are presented as number (percentage). Percentages are calculated based on available data
